# From the President’s desk: Part 3, 2025

**DOI:** 10.4102/safp.v67i1.6213

**Published:** 2025-08-31

**Authors:** Tasleem Ras

**Affiliations:** 1 Division of Family Medicine, Department of Family, Community and Emergency Care, Faculty of Health Sciences, University of Cape Town, Cape Town, South Africa; 2 South African Academy of Family Physicians, Durbanville, South Africa

Dear colleagues,

I was astonished to realize that half of 2025 had already passed! When I reflected on this realisation, I also pondered on the amount of work that has been completed, and the trying times that our country and health systems are facing.

In this reflection, I was reminded of the value inherent in our frontline staff in rural and urban primary care around the country. They perform their duties in circumstances of great need and poverty, and we must always be conscious of the personal price they and their families pay to ensure services are delivered to those in greatest need.

Members of the academy have been hard at work, advocating for family medicine, primary and district care, and for more community engagement. We have several exciting developments to report.

One of our strategic priorities was to progressively achieve financial sustainability. This would predominantly be not only through fully paid-up memberships but also by accessing other funding opportunities. To date, our membership has shown a substantial increase from 2024, although we are still short of the target membership that was decided at our annual strategic planning session in December 2024. Please sign up on our website and bring all your friends and colleagues along!

However, the academy has been busy providing a service to a national educational project that has generated income, which has given us some breathing space. We still provide a continuing professional development (CPD) accreditation service to industry and organisations, which is also contributing to our financial stability.

A very exciting development has been our ability to issue Section 18A certificates to donors, which qualify them to claim tax deductions from the South African Revenue Service (SARS). We are encouraging members and any readers to contribute via this route. Check out the links on our website for further information – every bit counts!

We are proudly celebrating that our national Treasurer, Prof. Mergan Naidoo (UKZN), was recently elected as the President of the South African Medical Association (SAMA). This promises to bring SAAFP and SAMA closer together, especially where our interests overlap. Another development is that we are discussing a *very* exciting possibility with our rural colleagues. Watch this space, and our social media platforms, for this wonderful announcement soon!

Our private sector colleagues have been making progress in the negotiations with funders to ensure that tariffs commensurate with our qualifications and that experience are allocated. While some of the larger medical aids have agreed to this, there are others who still need to be convinced. To this end, we are actively exploring ways to optimise this process, particularly because it appears that the State cannot supply the number of consultant positions required for employing all of our recent specialist graduates.

Our national conference, planned for 05–07 September 2025, with pre-conference workshops on 04 September, promises to be an event of note. The conference committee has secured great keynote speakers, while the scientific committee has put together a programme that is bursting with the pride of South African, and African, family medicine research.

I suppose the last announcement is that the SAAFP is 45-years-old this year! We are planning a celebration of our collective achievements, standing on the shoulders of giants. Here’s wishing us all well into the next 45 years!

